# Green‐Light‐Activated Photoreaction via Genetic Hybridization of Far‐Red Fluorescent Protein and Silk

**DOI:** 10.1002/advs.201700863

**Published:** 2018-03-12

**Authors:** Jung Woo Leem, Jongwoo Park, Seong‐Wan Kim, Seong‐Ryul Kim, Seung Ho Choi, Kwang‐Ho Choi, Young L. Kim

**Affiliations:** ^1^ Weldon School of Biomedical Engineering Purdue University West Lafayette IN 47907 USA; ^2^ Department of Agricultural Biology National Institute of Agricultural Sciences Rural Development Administration Wanju Jeollabuk‐do 55365 Republic of Korea; ^3^ Regenstrief Center for Healthcare Engineering Purdue University West Lafayette IN 47907 USA; ^4^ Purdue Quantum Center Purdue University West Lafayette IN 47907 USA

**Keywords:** fluorescent proteins, photocatalysis, photosensitization, reactive oxygen species, transgenic silk

## Abstract

Fluorescent proteins often result in phototoxicity and cytotoxicity, in particular because some red fluorescent proteins produce and release reactive oxygen species (ROS). The photogeneration of ROS is considered as a detrimental side effect in cellular imaging or is proactively utilized for ablating cancerous tissue. As ancient textiles or biomaterials, silk produced by silkworms can directly be used as fabrics or be processed into materials and structures to host other functional nanomaterials. It is reported that transgenic fusion of far‐red fluorescent protein (mKate2) with silk provides a photosensitizer hybridization platform for photoinducible control of ROS. Taking advantage of green (visible) light activation, native and regenerated mKate2 silk can produce and release superoxide and singlet oxygen, in a comparable manner of visible light‐driven plasmonic photocatalysis. Thus, the genetic expression of mKate2 in silk offers immediately exploitable and scalable photocatalyst‐like biomaterials. It is further envisioned that mKate2 silk can potentially rule out hazardous concerns associated with foreign semiconductor photocatalytic nanomaterials.

Visible light‐driven plasmonic photocatalysis, which relies on the combination of semiconductor photocatalysts with metal nanostructures/nanoparticles, has received considerable attention for solar energy conversion and utilization.^1^ Solar photocatalysis has a variety of energy and environmental applications, such as hydrogen generation, carbon dioxide reduction, desalination, disinfection, and water/air purification.[[qv: 1c,2]] Specifically, the production of reactive oxygen species (ROS) photoinduced from photocatalyis has direct utilization for environment remediation and biomedicine. However, such applications are often intrinsically limited for large‐scale and mass production. In addition, potentially hazardous and adverse (e.g., carcinogenic and cytotoxic) effects associated with semiconductor nanoparticles have limited the widespread utilization.^3^ In this respect, we take inspiration from nature to identify and characterize plasmonic photocatalyst‐like biological materials and further translate them into industrially relevant production processes.

The phototoxicity of fluorescent proteins, in particular red fluorescent proteins (RFP) is unanimously acknowledged in several different scientific communities; Some of RFPs generate and release ROS upon light excitation, while the exact types of ROS vary among different RFP variants.^4^ Since the use of such RFP was restricted by cytotoxicity, non‐cytotoxic RFP variants have been successfully developed for whole‐cell labeling and cellular imaging in vivo.^5^ In contrast, cytotoxic RFP has also been used as a means of selectively damaging specific proteins upon light activation, which is known as chromophore‐assisted light inactivation.^6^ In the latter case, RFP is recapitulated as “genetically encoded ROS‐generating proteins” for inactivating target cells and ablating tissue of interest.[[qv: 4d]] All of these characteristics of RFP suggest that semiconductor nanocrystals or conjugated nanoparticles for plasmonic photocatalysis can be replaced by phototoxic RFP.

Some fluorescent proteins participate in Type I and Type II photosensitization reactions.^7^ Predominant ROS generated by fluorescent proteins depends on the type of photosensitization reactions and the concentration of local molecular oxygen (i.e., electron acceptor). For example, (enhanced) green fluorescent protein, (E)GFP typically produces singlet oxygen (^1^O_2_) via Type II photosensitization reaction, in which energy transfer occurs from the excited triplet state of the fluorescent protein to molecular oxygen.^8^ RFP, such as KillerRed, can undergo Type I photosensitization reaction, in which electron transfer to molecular oxygen yields superoxide (O_2_^•−^).[[qv: 6c,9]] Another interesting aspect of ROS resulting from Type I and Type II photosensitization reactions is that the maximum migration (or damage) distance of O_2_^•−^ and ^1^O_2_ is less than 200–300 nm, depending on the surrounding environments.[[qv: 6a,b,10,11]] This relatively short damage distance can be advantageous as a safeguard, given that O_2_^•−^ and ^1^O_2_ are instantaneously reactive and toxic. Importantly, the resultant ROS (i.e., O_2_^•−^ and ^1^O_2_) generated by plasmonic photocatalysis using visible light is the same as that of RFP photosensitization reactions.[[qv: 6c,9,12]]

In this work, we introduce biological hybridization of far‐red fluorescent proteins and natural proteins (i.e., silk) for a new class of genetically encoded photosensitization that can be activated by visible (or solar) light, producing selective ROS in a similar manner of plasmonic photocatalysis. Direct detection of ROS is known to be highly challenging, because ROS is extremely reactive and unstable. Thus, we implement several different approaches using turn‐on/‐off fluorescent radical probes and physical quenchers/scavengers to experimentally validate ROS generated by transgenic RFP silk upon green light activation. We demonstrate that transgenic RFP silk can be mass‐produced by scalable and continuous manufacturing using the currently available textile infrastructure. Using the polymeric nature of silk, transgenic RFP silk is further processed into nanomaterials and nanostructures in a variety of forms. The use of plasmonic photocatalyst‐like proteins can overcome the limitation of potential adverse effects associated with foreign synthesized nanoparticles. We also envision that this bioreactor approach could potentially offer an alternative green manufacturing strategy for next generation photocatalysts.

We provide the impetus of visible light‐activated genetically encoded ROS‐generating multifunctional biomaterials, by exploiting silk containing recombinant RFP produced by transgenic silkworms (*Bombyx mori*) (**Figure** **1**a). Silk produced by silkworms has extensively been utilized as fabrics and processed into engineered biomaterials due to its various merits of the superior mechanical and optical properties as well as the biocompatibility and biodegradability.^13^ In particular, we take advantage of genetically engineered domesticated silkworms; transgenes of interests are expressed by germline transformation using the gene splicing method *piggyBac*.[[qv: 13f,14]] This silkworm transgenesis method can yield transformed animals with multiple successive generations and can produce recombinant substances in large amounts. The manufacturing processes of photocatalytic semiconductor nanoparticles often involve negative environmental consequences.^3^ On the other hand, silkworm transgenesis enables us to readily produce natural photocatalysts and photosensitizers in an eco‐friendly manner, minimizing the use of industrial facilities. Regarding an ecological hazard, it is highly unlikely that transgenic silkworms pose threats to natural ecosystems, because silkworms are dependent on humans for survival and reproduction as a completely domesticated indoor insect.

**Figure 1:**
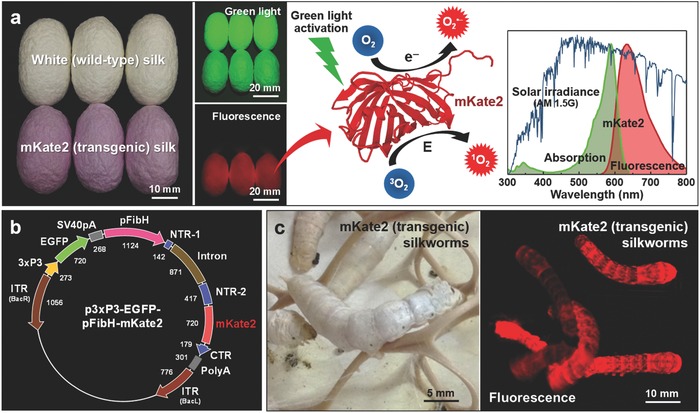
Genetically encoded hybridization of far‐red fluorescent protein (mKate2 and PDB ID: 3BXB) and silk for plasmonic photocatalysis‐like photosensitization. a) Schematic illustration of reactive oxygen species (ROS)‐generating mKate2 (transgenic) silk under green light activation. Superoxide (O_2_^•−^) and singlet oxygen (^1^O_2_) are generated by mechanisms of electron (e^−^) transfer and energy (E) transfer, respectively. Photographs of white (wild‐type) and mKate2 (transgenic) silk cocoons and fluorescent image of mKate2 silk cocoons. Green light belongs to the peak wavelength range of the solar spectrum. b) Construction of transfer vector p3xP3‐EGFP‐pFibH‐mKate2 for mKate2 silkworm transgenesis. c) Photograph and fluorescent image of mKate2 (transgenic) silkworms.

We choose mKate2, which is a far‐red monomeric fluorescent protein.[[qv: 5a]] From a phototoxicity standpoint, mKate and mKate2 are widely considered as one of the cytotoxic standards.[[qv: 5b–d]] From a protein structural standpoint, the phototoxic action of mKate is commonly acknowledged to originate from a cleft‐like opening channel filled with water molecules inside, allowing for enhanced generation and release of ROS. Specifically, mKate has a cleft‐like β‐barrel frame between β sheets (β7 and β10), resulting in relatively high phototoxicity.[[qv: 4e,15]] Several other fluorescent proteins, including KillerRed,^16^ SuperNova,[[qv: 6c]] KillerOrange,^17^ Dronpa,^18^ TurboGFP,^19^ and mCherry,^20^ have a similar β‐barrel structure with a water‐filled pore, which can also be used to tune the excitation wavelength range and to select the photosensitization properties. For the hybridization of mKate2 and silk, mKate2 gene is fused with N‐terminal and C‐terminal domains of the fibroin heavy chain promoter (pFibH); p3xP3‐EGFP‐pFibH‐mKate2 is the constructed transformation vector (Figure 1b and Figures S1 and S2 (Supporting Information)). 3xP3‐EGFP is served only for screening a large number of G1 broods, because EGFP fluorescent signals are easily monitored in the stemmata and the nervous system at early embryonic and larval stages. The silk gland of genetically encoded mKate2 silkworms is red fluorescent (Figure 1c and Figure S3 (Supporting Information)). The homogenous production of mKate2 silk results in a mass density of ≈12.6% mKate2/Fibroin H‐chain fusion recombinant protein.[[qv: 14e]] In Figure 1a, white (wild‐type) silk cocoons are not fluorescent, while mKate2‐expressing silk cocoons are red fluorescent under green light excitation (Figure S4, Supporting Information).

In **Figure** **2**a, we photometrically analyze the photocatalytic activity of mKate2 silk by degrading organic blue dye molecules (i.e., methylene blue) in an aqueous solution under green laser light activation (λ_ex_ = 532 nm and optical intensity ≈0.2 mW mm^−2^; Supporting Information) at the ambient room temperature. Although this crude method is not specific to particular types of ROS, the photodegradation of methylene blue serves as a standard for validating photocatalysis. However, silk has a strong affinity to organic molecules and metal ions.[[qv: 14e,21]] Thus, the loss of blue color in a methylene blue solution containing mKate2 silk is attributable to the infiltration (i.e., adsorption) of methylene blue to silk fibers as well as the photolysis of methylene blue itself by green light. In this respect, we performed separate degradation measurements to account for the adsorption of methylene blue to silk under a dark condition (i.e., no light irradiation) and the photolysis of methylene blue without any silk disks (Figure S5, Supporting Information). After factoring out these confounding effects, the contribution of ROS generated by the mKate2 silk is significant; a linear fit between ln(*C_t_*/*C*_0_) of methylene blue by mKate2 silk and the irradiation time *t* results in an apparent pseudo‐first‐order rate constant (*k*_app_) value of 2.46 × 10^−4^ min^−1^ (inset of Figure 2a).

**Figure 2:**
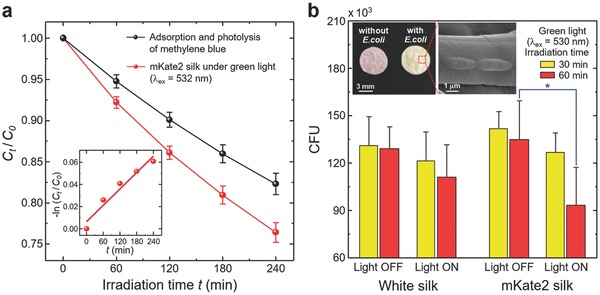
Photocatalytic activity of mKate2 silk for degrading methylene blue and inactivating bacteria under green light activation at ambient temperature. a) Photodegradation of methylene blue in aqueous solutions by mKate2 silk under green laser irradiation. (Inset) Kinetic plot for methylene blue photodegradation by mKate2 silk after factoring out both adsorption and photolysis of methylene blue. *C_t_*/*C*_0_ is a relative concentration of methylene blue in an aqueous solution, where *C*_0_ and *C_t_* are the concentrations of methylene blue before and after green light irradiation, respectively. The error bars are standard deviations. b) Colony‐forming units (CFU) of live *E. coli* (DH5α) are counted in white silk and mKate2 silk disks with and without weak green LED light activation for different irradiation periods of 30 and 60 min. (Insets) Representative photograph of mKate2 silk disks with and without *E. coli* and scanning electron microscopy (SEM) image of mKate2 silk attached with *E. coli* before light irradiation. Statistically significant reduction in the survival of *E. coli* occurs between 60 min irradiated (Light ON) and un‐irradiated (Light OFF) mKate2 silk (multiple comparison *p* value = 0.031). The error bars represent standard deviations from three assays with four replicates (12 samples) in each group.

As a model system of testing ROS production, we also examine the phototoxicity of mKate2 silk on *Escherichia coli* upon green light activation (Figure 2b). Historically, ROS generated by conventional photocatalysis has extensively been validated by demonstrating their antimicrobial activities.^22^ After DH5*α E. coli* cells are attached on silk disks (inset of Figure 2b), illumination from an easily accessible green light‐emitting diode (LED) source (λ_ex_ = 530 nm with a full width at half maximum (FWHM) of 30 nm and optical intensity ≈ 0.02 mW mm^−2^; Supporting Information), which is ≈10 times weaker than that of the green laser source above, is irradiated on the surface of white silk and mKate2 silk for 30 and 60 min at the ambient room temperature. Such green light activation is not only accessible from sunlight, but also belongs to the peak solar radiation spectrum. Dark controls are also maintained without any light irradiation. Colony‐forming unit (CFU) counts show a statistically significant difference only in bacterial inactivation between irradiated (Light ON) and un‐irradiated (Light OFF) mKate2 silk for 60 min (multiple comparison *p* value = 0.031) (Tables S1 and S2, Supporting Information). The survival rate of *E. coli* from mKate2 silk under weak green light activation (Light ON) is reduced to 45%, compared with the corresponding dark controls (Light OFF). This result supports the idea of green light‐activated genetically encoded photosensitization as an alternative ROS generation route, completely avoiding the use of photocatalytic semiconductor nanoparticles.

We further investigate specific types of ROS produced by mKate2 silk upon green light activation (*λ*_ex_ = 532 nm and optical intensity ≈ 0.2 mW mm^−2^) (**Figure** **3**). First, we detect O_2_^•−^ generated by mKate2 silk via primarily Type I photosensitization reaction. The generation and release of O_2_^•−^ are monitored using fluorescent radical probes; 4‐[(9‐acridinecarbonyl)amino]‐2,2,6,6‐tetramethylpiperidin‐1‐oxyl (TEMPO‐9‐ac) is commonly used to sense O_2_^•−^.^9^ Under consistent green light irradiation on mKate2 silk disks immersed in TEMPO‐9‐ac solutions, fluorescent signals of TEMPO‐9‐ac (λ_ex_ ≈ 360 nm and λ_em_ ≈ 440 nm) are detected in two different configurations (Figure S6, Supporting Information): (i) The turn‐on fluorescent radical probes on the mKate2 silk surface are diffused in the TEMPO‐9‐ac solution. In Figure 3a, the fluorescent emission intensity of TEMPO‐9‐ac increases monotonously with the duration of green light irradiation, compared with the baseline signals before light activation (controls). (ii) After TEMPO‐9‐ac is permeated into the silk disks, the turn‐on fluorescent radical probes remain inside, which in turn emit blue fluorescence of TEMPO‐9‐ac from the mKate2 silk disks. 240 min irradiation of green light leads to a twofold increase in the radical probe fluorescent intensity from the silk disks infiltrated with TEMPO‐9‐ac, compared with the un‐irradiated mKate2 silk disks (controls) (Figure S7, Supporting Information). These results are in excellent agreement with O_2_^•−^ released from KillerRed, which is one of the highly phototoxic RFP variants.^9^ Second, we detect ^1^O_2_ generated by mKate2 silk via Type II photosensitization reaction under the same green light activation, using 9,10‐anthracenediyl‐bis(methylene)dimalonic acid (ABDA) as a radical probe. While the original state of ABDA emits fluorescence (λ_ex_ ≈ 380 nm and λ_em_ ≈ 431 nm), ABDA reacts with ^1^O_2_ to yield endoperoxide as a turn‐off fluorescent radical probe, reducing its fluorescent intensity.^23^ In Figure 3b, the intensity of ABDA fluorescent peaks gradually drops as the irradiation time increases, supporting the generation of ^1^O_2_.

**Figure 3:**
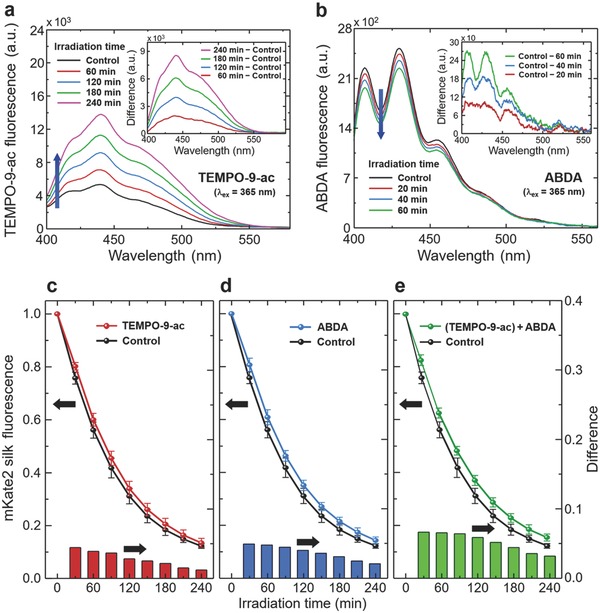
Turn‐on/off fluorescence detection and fluorogenic scavenger detection of ROS generated by mKate2 silk upon green light activation. a,b) Fluorescent emission signals of radical probes are recorded from solutions containing mKate2 silk disks. (a) O_2_^•−^ mediated by Type I photosensitization reaction, captured by turn‐on fluorescent signals of TEMPO‐9‐ac. (b) ^1^O_2_ mediated by Type II photosensitization reaction, detected by reduction of the original ABDA fluorescence. (Insets) Difference in fluorescent spectra with respect to controls before green light activation. c–e) Reduction in photobleaching of mKate2 silk disks is quantified by the normalized fluorescent intensity of mKate2 silk in the presence of fluorogenic scavengers of TEMPO‐9‐ac for O_2_^•−^ (c), ABDA for ^1^O_2_ (d), and a mixture of TEMPO‐9‐ac and ABDA (e). As a control, the normalized fluorescent intensity of mKate2 silk without the fluorogenic scavengers is plotted in black. The error bars are standard deviations. (Bottom insets) Differences in fluorescent intensity with respect to the control.

Using fluorogenic scavengers, we additionally validate the generation of O_2_^•−^ and ^1^O_2_ from Type I and Type II photosensitization reactions of mKate2 silk. The phototoxicity of RFP is always accompanied by photobleaching, because the formation of ROS itself facilitates the degradation of RFP excitation–emission cycles.^24^ Interestingly, TEMPO‐9‐ac and ABDA, which are fluorescent radical probes, can also be used as physical quenchers of O_2_^•−^ and ^1^O_2_, respectively, without directly reacting with other free radicals (Figure S8, Supporting Information).^25^ In Figure 3c,d, the inhibition of TEMPO‐9‐ac and ABDA in photobleaching of mKate2 silk provides another level of evidence, supporting O_2_^•−^ and ^1^O_2_ production. In other words, the uptake of local surrounding ROS (O_2_^•−^ and ^1^O_2_) prevents mKate2 silk from being photodamaged, which is manifested by the relatively sustained fluorescent intensity of mKate2 silk. In a mixed solution of TEMPO‐9‐ac and ABDA, the fluorescent emission of mKate2 silk is further maintained (Figure 3e). We also confirm reduced photobleaching of mKate2 silk using other scavengers of O_2_^•−^ (nitro blue tetrazolium chloride, NBT) and ^1^O_2_ (sodium azide, NaN_3_) (Figure S9, Supporting Information).^26^

The direct use of silk fibers produced by silkworms has its own advantage as utilized in the textile industry, because the transgenic silk has the comparable mechanical properties to wild‐type silk to weave fabrics (Figure S10, Supporting Information). Silk fibroin can further be processed into polymeric materials for fabricating artificially engineered biomaterials and optical materials in a variety of forms with biocompatibility and bioabsorbability.[[qv: 13b–f]] However, the conventional fibroin processing methods are inappropriate for mKate2 silk,[[qv: 13c–e]] because fluorescent proteins are highly susceptible to denaturation from high temperature and pH values.[[qv: 13f,27]] In our case, to minimize heat‐induced denaturation of mKate2, mKate2 silk fibroin is extracted from silk cocoons at low temperature of 45 °C, assisted by alcalase enzyme and dithiothreitol (DTT) treatments.[[qv: 13f]] A reductase, such as DTT, is beneficial for renaturing the protein structure by reducing the disulfide bonds of proteins and peptides in a solvent.^28^ In **Figure** **4**a–c, mKate2 silk fibroin is processed into an aqueous solution and then is formed into a flexible thin film. The fluorescent property of mKate2 is maintained in both of the regenerated mKate2 silk solution and the regenerated mKate2 silk film under green light excitation (Figure S11, Supporting Information). Importantly, the generation of O_2_^•−^ and ^1^O_2_ from the regenerated mKate2 silk products is also detected using TEMPO‐9‐ac and ABDA, respectively (Figure 4d,e). With prolonged green light irradiation (λ_ex_ = 532 nm and optical intensity ≈ 0.2 mW mm^−2^), the fluorescent signal of TEMPO‐9‐ac increases, while that of ABDA decreases, supporting the two types of ROS generation. Similarly, the photodegradation of methylene blue by the mKate2 silk film results in *k*_app_ = 1.12 × 10^−3^ min^−1^ under green light irradiation, after factoring out the confounding effects (i.e., adsorption and photolysis of methylene blue) (Figure S12, Supporting Information).

**Figure 4:**
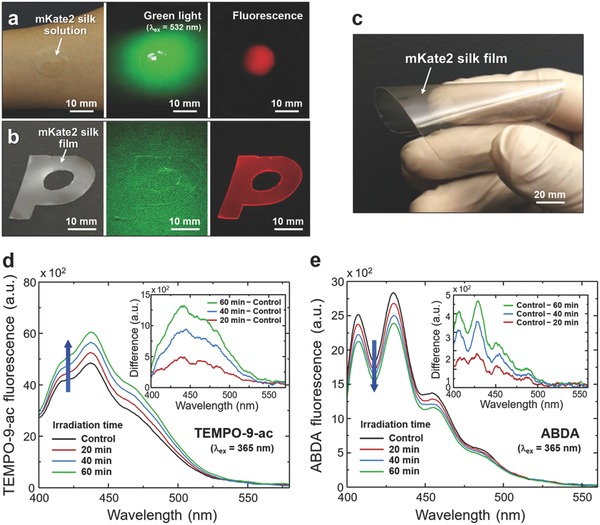
Regenerated mKate2 silk and detection of ROS generation upon green light activation. a,b) Photographs and fluorescent images of mKate2 silk solution and film. c) Photograph of large‐area flexible mKate2 silk film with a diameter of 120 mm. d,e) For regenerated mKate2 silk films, fluorescent emission signals of radical probes of TEMPO‐9‐ac for O_2_^•−^ (d) and ABDA for ^1^O_2_ (e). (Insets) Differences in fluorescent spectra with respect to controls before green light activation.

From an ecological standpoint, our results may suggest that the primary purpose of fluorescent proteins in nature could be photoinducible ROS generation, while the fluorescent emission may be a secondary consequence. From a mechanistic standpoint, ROS generation from fluorescent proteins is known to involve long‐range electron transfer via two possible mechanisms of direct tunneling and hopping inside fluorescent proteins.[[qv: 4c,29]] The current understanding of this mechanism is based on quantum mechanics, because electron tunneling over such a long distance of 1.5–3 nm is typically impossible in vacuum.^30^ From an electron donor standpoint, (E)GFP has been successfully tested for generating electricity as photodetectors and photovoltaics for bioenergy applications.^31^ From a photocatalysis standpoint, the direct photosensitization properties of RFP have not yet been exploited for scalable photoreaction in a similar manner of plasmonic photocatalysis.

In conclusion, the reported hybridization of mKate2 and silk using genetically engineered silkworms can offer several pivotal advantages. Without a need of additional nanoconjugations (e.g., metals, dye molecules, and quantum dots), RFP can be excited by solar (visible) and green light, avoiding the most common carcinogen exposure of ultraviolet light. Both fluorescent proteins and silk are degradable and digestible,^32^ eliminating the potential risk of exposure and consumption. As a biosynthesis reactor (i.e., green manufacturing), silkworm transgenesis is well‐established for producing recombinant proteins in large amounts.[[qv: 13f,14]] As ancient textile materials, silk fibers are easily woven into large‐area, continuous, and flexible fabrics using the existing textile manufacturing infrastructure.[[qv: 14d,e]] The unprecedentedly strong light scattering of native silk, which is manifested as the “silvery” and “lustrous” reflection,^33^ can enhance interactions of light with RFP inside silk fibers.

## Experimental Section

*Removal of Sericin in Silk (i.e., Degumming)*: For effective generation and release of ROS from mKate2 silk, it was critical to remove the outermost layer (i.e., sericin) of silk fibers. Sericin was removed using a degumming process. The outer sericin layer is commonly removed to improve the color, sheen, and texture of silk in the silk textile industry. However, conventional sericin removal methods are inappropriate for mKate2 silk, because these involve a boiling process in an aqueous solution.[[qv: 13c–e]] In this case, mKate2 silk cocoons were soaked in a prewarmed mixture solution of sodium carbonate (Na_2_CO_3_, 0.2%) and Triton X100 (0.1%) at low temperature of <60 °C under a vacuum pressure. During the degumming process, low pressure treatments (620 mmHg) were repeated several times to uniformly infiltrate the solution between silk fibers to remove most sericin. The degummed mKate2 silk cocoons were dried in dark under the ambient air conditions.

*Photodegradation of Methylene Blue as General Photocatalytic Quantification*: Photodegradation of methylene blue, resulting from ROS generated by mKate2 silk under green light activation, was quantified. For mKate2 silk specimens, silk cocoons were punched into 5 mm diameter disks with a thickness of ≈400 µm. Methylene blue solutions (1 mL 0.05 wt% methylene blue in 14 mL deionized water) containing 12 silk disks (total weight = 0.06 g) were prepared. To reach the adsorption–desorption equilibrium in each test, the silk disks were stirred with 400 rpm in dark for 2 h. Then, the silk disks were irradiated by green light (λ_ex_ = 532 nm and optical intensity ≈ 0.2 mW mm^−2^) for 4 h, while being stirred. Aliquots (0.5 mL) were collected repeatedly with a fixed time interval and the spectral absorption of methylene blue was measured using a fiber bundle‐coupled spectrometer with a white‐light tungsten halogen source. To exactly quantify the photocatalytic activity of mKate2 silk only, separate degradation tests of methylene blue were also carried out to factor out two confounding effects: (i) the adsorption of methylene blue to silk under a dark condition (i.e., no light irradiation) and (ii) the photolysis of methylene blue without any silk disks by green light. For each elapsed irradiation time, a relative concentration *C_t_*/*C*_0_ of methylene blue was calculated using the absorption spectrum peak values *C_t_* at λ = 668 nm normalized by the absorption value *C*_0_ before light irradiation (Figure 2a and Figure S5 (Supporting Information)). The reaction kinetics were estimated, following the apparent pseudo‐first‐order rate equation of Langmuir–Hinshelwood kinetics: ln(*C_t_*/*C*_0_) = −*k*_app_ *t*, where *k*_app_ is the rate constant (min^−1^) and *t* is the irradiation time (insets of Figure 2a).

*Bacterial Inactivation as General Detection of ROS*: ROS generated by mKate2 silk was tested to inactivate *E. coli* upon green light irradiation. Four different groups of two different types of silk (i.e., white silk and mKate2 silk) and two light conditions (i.e., irradiation and un‐irradiation) were conducted. These experiments were repeated for two different irradiation times of 30 and 60 min. Each bacterial inactivation experiment was performed in three assays with four replicates (*n* = 12) in each group for statistical analyses. DH5*α E. coli* cells were grown in a Luria–Bertani (LB) medium at 37 °C in a shaking incubator to an optical density at 600 nm (OD_600_) of 2.5 (≈2 × 10^9^ cells mL^−1^). The culture was diluted tenfold and subsequently white silk and mKate2 silk disks (diameter = 6 mm) were placed on the culture. After incubation at 37 °C for 60 min, each silk disk was dried in dark for 30 min. For optical excitation of mKate2, the silk disks on a hydrated filter paper were irradiated with the green LED source (λ_ex_ = 530 nm with a FWHM of 30 nm and optical intensity ≈0.02 mW mm^−2^) for 30 and 60 min at the ambient room temperature, including white silk disks for comparisons. Without any irradiation, both white silk and mKate2 silk disks were kept in dark under the same conditions as two different control groups. After green light activation, each silk disk was transferred to a phosphate buffered saline (PBS; pH 7.4) solution (1 mL) and *E. coli* cells were eluted by shaking incubation for 60 min. To achieve a reasonable number of surviving cells for counting the colonies, the eluted cells were diluted up to 1000‐fold, were plated on the LB agar, and were incubated overnight at 37 °C. CFU from the mKate2 silk disk irradiated under weak green light for 60 min was clearly lower than that of the mKate2 silk disk in dark (Figure 2b). Because the biological experiments were carried in four different groups, analysis of variance (ANOVA) and multiple comparisons tests were conducted. In particular, Duncan multiple comparison (two‐sided) tests set a 5% level of significance for all pairs of means (six possible comparisons). The statistical analyses were performed using Stata 14.2 (College Station, TX, USA).

*Detection of Superoxide (O_2_^•−^) and Singlet Oxygen (^1^O_2_) Using Fluorescent Radical Probes*: As free radical probes of O_2_^•−^ and ^1^O_2_, TEMPO‐9‐ac and ABDA were used, respectively. In the original state of TEMPO‐9‐ac, acridine is quenched in the presence of nitroxide moiety. O_2_^•−^ converts nitroxide to the corresponding piperidine, which eliminates the quenching of the blue fluorophore. Thus, blue fluorescent emission from acridine appears under ultraviolet light excitation (λ_ex_ ≈ 360 nm and λ_em_ ≈ 440 nm).^9^ The original state of ABDA emits fluorescence under ultraviolet light excitation (λ_ex_ ≈ 380 nm and λ_em_ ≈ 431 nm).^23^ After ABDA reacting with ^1^O_2_, it is converted to an endoperoxide form that leads to a decrease in the fluorescent intensity. In this study, TEMPO‐9‐ac and ABDA were initially dissolved in dimethyl sulfoxide and were diluted in PBS, respectively, resulting in each solution containing TEMPO‐9‐ac (20 × 10^–6^ m) or ABDA (20 × 10^–6^ m). In each measurement, 12 silk disks (diameter = 5 mm and total weight = 0.06 g) or regenerated silk films were immersed in a TEMPO‐9‐ac or ABDA solution with stirring of 400 rpm. Because water‐soluble molecules were easily smeared inside silk fibers, the adsorption–desorption equilibrium was achieved prior to green light activation; the silk disks were kept in the solution with stirring of 400 rpm in dark for 2 h at least. Turn‐on fluorescent signals of TEMPO‐9‐ac solutions and turn‐off fluorescent signals of ABDA solutions were spectrofluorimetrically monitored using a spectrometer. Turn‐on fluorescence (i.e., TEMPO‐9‐ac) from the mKate2 silk disks was also imaged and was measured using a custom‐build mesoscopic (between microscopic and macroscopic) imaging setup (Figure S7, Supporting Information).[[qv: 14e,34]]

*Detection of Superoxide (O_2_^•−^) and Singlet Oxygen (^1^O_2_) Using Scavengers*: By detecting reduced photobleaching of mKate2 silk in the presence of O_2_^•−^ and ^1^O_2_ scavengers (ROS contributed to photobleaching^24^), Type I and Type II photosensitization reactions were further validated. In particular, advantage of TEMPO‐9‐ac and ABDA as fluorogenic scavengers (i.e., physical quenchers) of O_2_^•−^ and ^1^O_2_ was taken, respectively (Figure S8, Supporting Information).^25^ Under green light irradiation (λ_ex_ = 532 nm and optical intensity ≈ 0.2 mW mm^−2^), the photobleaching effect of mKate2 silk was reduced in the presence of TEMPO‐9‐ac (20 × 10^–6^ m); the fluorescent emission was relatively maintained over the irradiation time in the presence of the physical scavenger of O_2_^•−^. Similarly, ^1^O_2_ generation was detected by the maintained fluorescent intensity of mKate2 silk in the presence of ABDA (20 × 10^–6^ m). In addition, reduced photobleaching rates of mKate2 silk were confirmed using NBT (200 × 10^–6^ m) and NaN_3_ (200 × 10^−3^ m), which are often used as a scavenger of O_2_^•−^ and ^1^O_2_, respectively (Figure S9, Supporting Information).^26^

*Regeneration of mKate2 Silk*: To use the polymeric nature of silk, mKate2 silk was regenerated by extracting mKate2 silk fibroin from silk cocoons. mKate2 silk cocoons were cut to pieces with sizes less than 5 mm and were heated for 4 h at ≈45 °C in a aqueous solution of NaHCO_3_ (50 × 10^−3^ m) with alcalase (1.5 mL L^−1^) with stirring of 400 rpm. Subsequently, the silk fibers were washed with deionized water (≈35 °C) several times and were dried in dark under the ambient conditions for 24 h. It was also noted that conventional fibroin dissolution methods are not ideal for mKate2 silk, because these require chemical‐based solution treatments at temperature of 60 °C.[[qv: 13c–e]] In this case, the silk fibers were completely dissolved in a lithium bromide (LiBr, 9.5 m) solution with DTT (1 × 10^−3^ m) at 45 °C. The dissolved solution was filtered through a miracloth and was dialyzed with deionized water for 2 d to remove the remaining salt. The final concentration of mKate2 silk fibroin in the solution was ≈4–5% (w v^−1^). When the same method was followed for wild‐type white silk under the identical conditions, a similar final concentration of silk fibroin was obtained. The solution was stored at 4 °C in dark before use. The fabrication process of the mKate2 silk solution was carried out in dark environment to minimize photobleaching of mKate2 in silk by the room light. To form silk films, the solution was dried at 30 °C for 12 h in an oven.

## Conflict of Interest

The authors declare no conflict of interest.

## Supporting information

Supplementary
