# Supplementary material for: Green‐Light‐Activated Photoreaction via Genetic Hybridization of Far‐Red Fluorescent Protein and Silk
Source: Adv Sci (Weinh). 2018 Mar 12;5(6):1700863. doi: 10.1002/advs.201700863 (PMC6010726; doi:10.1002/advs.201700863)
Supplement: Supplementary file 1 — Supplementary [file ADVS-5-1700863-s001.pdf]

Copyright WILEY-VCH Verlag GmbH & Co. KGaA, 69469 Weinheim, Germany, 2018.

ADVANCED SCIENCE Open Access

Supporting Information

for *Adv. Sci.,* DOI: 10.1002/advs.201700863

Green-Light-Activated Photoreaction via Genetic Hybridization of Far-Red Fluorescent Protein and Silk

Jung Woo Leem, Jongwoo Park, Seong-Wan Kim, Seong-Ryul Kim, Seung Ho Choi, Kwang-Ho Choi, and Young L. Kim**

Copyright WILEY-VCH Verlag GmbH & Co. KGaA, 69469 Weinheim, Germany, 2018.

Supporting Information

Green Light-Activated Photoreaction via Genetic Hybridization of Far-red Fluorescent Protein and Silk

Jung Woo Leem, Jongwoo Park, Seong-Wan Kim, Seong-Ryul Kim, Seung Ho Choi, Kwang-Ho Choi, and Young L. Kim

Materials

For silkworm transgenesis for producing mKate2 silk, we used *Bombyx mori* bivoltine strain, Keumokjam (F1 hybrid between the Japanese parental line *Jam 125* and the Chinese parental line *Jam 140*) from the National Institute of Agricultural Sciences (Wanju, Republic of Korea). DNA-injected eggs were kept at 25 °C in moist Petri dishes. The hatched larvae (i.e. silkworms) were reared in groups and fed with mulberry leaves under standard conditions (e.g. 25 ± 2 °C and 80 ± 10% relative humidity). For wild-type white silk, *Bombyx mori* (Baekokjam, *Jam 123* × *Jam 124*) was used.

We used the following chemicals as received: alcalase enzyme, dialysis tube (pore size 12,000 Da MWCO), dimethyl sulfoxide (DMSO; $(CH_3)_2SO$, 99%), dithiothreitol (DTT; $C_4H_{10}O_2S_2$, ≥ 98%), lithium bromide (LiBr, ≥ 99%), nitro blue tetrazolium chloride (NBT; $C_{40}H_{30}Cl_2N_{10}O_6$, ≥ 98%), methylene blue ($C_{16}H_{18}ClN_3S$, 0.05 wt.% in H_2O), miracloth (pore size 22 – 25 μm), phosphate buffered saline (PBS; pH 7.4), sodium azide (NaN_3, ≥ 99.5%), sodium carbonate (Na_2CO_3, ≥ 99%), Triton X100, and 9,10-anthracenediyl-bis(methylene)dimalonic acid (ABDA; $C_{22}H_{18}O_8$, ≥ 90%) were purchased from Sigma-Aldrich Co. (Milwaukee, USA). 4-[(9-acridinecarbonyl)amino]-2,2,6,6-tetramethylpiperidin-1-oxyl (TEMPO-9-ac; $C_{23}H_{26}N_3O_2$, 95%) was purchased from Synchem UG & Co. KG

(Altenburg, Germany). De-ionized (DI) water (Milli-Q® system) was used. All experiments were performed under the ambient conditions (22 ± 2 °C and 40 ± 10% relative humidity).

Construction of plasmid vector DNA for silk transgenesis

We constructed the transition vector p3xP3-EGFP-pFibH-mKate2 as the *piggyBac*-derived vector and injected the vector DNA with a helper vector into pre-blastoderm embryos, as shown in the construction sequence map (Figures S1 and S2). To obtain the fibroin pormoter, the DNA fragment (GenBank Accession No. AF226688, nucleotides 61312–63870) including pFibH promoter domain (1124 bp), N-terminal region 1 (NTR-1, 142 bp), first intron (871 bp), and N-terminal region 2 (NTR-2, 417 bp) was amplified by polymerase chain reaction (PCR) using the genomic DNA from *Bombyx mori* and specific primers (pFibHN-F: 5′-GGCGCGCCGTGCGTGATCAGGAAAAAT-3′ and pFibHN-R: 5′-TGCACCGACTGCAGCACTAGTGCTGAA-3′), followed by treatments with restriction enzymes of *Asc*I/*Not*I. The resultant DNA fragment was cloned into pGEM-T Easy Vector System (Promega, Co), named as pGEMT-pFibH-NTR. The DNA fragment (GenBank Accession No. AF226688, nucleotides 79021–79500) including C-terminal region (179 bp, CTR) and poly(A) signal region (301 bp) of the heavy chain was amplified by PCR using genomic DNA from *Bombyx mori* and specific primers (pFibHC-F: 5′-CCTGCAGGAAGTCGACAGCGTCAGTTACGGAGCTGGCAGGGGA-3′ and pFibHC-R: 5′- GGCCGGCC TATAGTATTCTTAGTTGAGAAGGCATA -3′) and then the resultant DNA fragment was cloned into pGEM-T Easy Vector System with the restriction enzymes of *Sal*I/*Sbf*I/*Fse*I, named as pGEMT-CTR. These two fragments were cloned with pBluescriptII SK(-) (Stratagene, CA) digested with *Apa*I/*Sal*I, creating pFibHNC-null. The mKate2 gene was synthesized from BIONEER Co., and then it was cloned into pGEM-T Easy Vector System pGEMT-mKate2 (720 bp). N- and C-terminal had the *Not*I and *Sbf*I restriction sites, respectively. The mKate2 cDNA was digested with *Not*I/*Sbf*I and was subcloned into a

pFibHNC-null digested with *Not*I/*Sbf*I, resulting in pFibHNC-mKate2. The pFibHNC-mKate2 vector was digested with *Asc*I/*Fse*I and was subcloned into pBac-3xP3-EGFP. The resultant vector was named as p3xP3-EGFP-FibH-mKate2.

Light sources for green light irradiation

For optical excitation of mKate2 silk, we used two green light sources with different optical intensity: i) A diode-pumped solid-state laser coupled with a 10× zoom Galilean beam expander was used (λ = 532 nm and optical intensity ≈ 0.2 mW mm^{-2} on the sample surface). ii) As an easily accessible common light source, a green light-emitting diode (LED) was used (λ = 530 nm with a FWHM of 30 nm and optical intensity ≈ 0.02 mW mm^{-2} on the sample surface).

Scanning electron microscopy and fluorescence confocal microscopy

We imaged the surface morphologies of silk cocoons using a scanning electron microscopy (SEM) system (FEI Quanta 3D FEG; Oregon, USA) at 10 keV. Exploiting the fluorescent emission of mKate2 silk, we performed confocal imaging using an Olympus Fluoview FV1000 confocal laser scanning system adapted to an Olympus IX81 inverted microscope with a 20× UPlanSApo water immersion objective (Olympus, Tokyo, Japan). In this system, a green laser excitation source (λ_{ex} = 543 nm) was used with a detection bandpass of 600 – 700 nm. The typical configuration of confocal microscopy can be summarized as follows: confocal aperture size = 50 μm (i.e. ~ 0.5 airy unit), NA = 0.4, and scan speed (pixel dwell time) = 10 μs $pixel^{-1}$. 43 image slices were stacked with a slice thickness of 5 μm along the z-axis, covering an area up to ~ 1270 μm × 1270 μm. The three-dimensional (3D) stacked image was visualized using Imaris 5.0.

Measurements of mechanical properties

To evaluate the basic mechanical properties of mKate2 silk fibers, we used a universal electromechanical test machine 100P/Q (TestResources Inc.) with a gauge length of 10 mm and an extension rate of 1 mm min^{-1} under ambient conditions. For both white silk and mKate2 silk fibers, we tested at least 10 randomly selected single fibers from three different cocoons. As shown in Figure S10a, mKate2 silk fibers exhibited no considerable change in the mechanical properties, such as the maximum strain, the maximum stress, and the Young's modulus (p-value = 0.4). Thus, mKate2 silk fibers can be treated as conventional silk fibers that can be woven or constructed into large-area and continuous fabrics (e.g. knitted dress and suit) using the textile technologies (Figure S10b).

Photodegradation of methylene blue by regenerated mKate2 silk films

We validated the photodegradation of methylene blue by mKate2 silk films under the green light activation (λ_{ex} = 532 nm and optical intensity ≈ 0.2 mW mm^{-2}) (Figure S12). For each elapsed irradiation time, a relative concentration C_t/C_0 of methylene blue was calculated using the absorption spectrum peak values C_t at λ = 668 nm normalized by the absorption value C_0 before light irradiation. We estimated the reaction kinetics, following the apparent pseudo-first-order rate equation of Langmuir-Hinshelwood kinetics: $\ln(C_t/C_0) = -k_{app}t$, where k_{app} is the rate constant (min^{-1}) and t is the irradiation time. After factoring out both adsorption and photolysis of methylene blue, the mKate2 silk films also showed the photocatalytic activity, resulting in the rate constant k_{app} value of 1.12×10^{-3} min^{-1} (Inset of Figure S12a).

Table S1

Multiple comparison tests of white silk and mKate2 silk with and without weak green LED light activation (irradiation time = 30 minutes)

Colony forming unit (CFU)	Mean difference	*t*	*p*-value	95% CI	
White silk + Light OFF[a] vs. White silk + Light ON[b]	-9,683	-0.77	0.472	-3,6252	16,885
mKate2 silk + Light OFF vs. White silk + Light ON	10,692	0.85	0.398	-14,573	35,957
mKate2 silk + Light ON vs. White silk + Light ON	-4,317	-0.34	0.732	-29,582	20,948
mKate2 silk + Light OFF vs. White silk + Light ON	20,375	1.63	0.145	-7,049	47,799
mKate2 silk + Light ON vs. White silk + Light ON	5,367	0.43	0.671	-19,898	30,632
mKate2 silk + Light OFF vs. mKate2 silk + Light ON	-15,008	-1.2	0.266	-41,577	11,560

[a]without green LED light irradiation.
[b]with green LED light irradiation.

Table S2

Multiple comparison tests of white silk and mKate2 silk with and without weak green LED light activation (irradiation time = 60 minutes)

Colony forming unit (CFU)	Mean difference	*t*	*p*-value	95% CI	
White silk + Light OFF[a)] vs. White silk + Light ON[b)]	-17,967	-1.04	0.305	-52,859	16,926
mKate2 silk + Light OFF vs. White silk + Light ON	5,884	0.34	0.736	-29,009	40,776
mKate2 silk + Light ON vs. White silk + Light ON	-35,850	-2.07	0.055	-72,543	843
mKate2 silk + Light OFF vs. White silk + Light ON	23,850	1.38	0.201	-12,843	60,543
mKate2 silk + Light ON vs. White silk + Light ON	-17,883	-1.03	0.307	-52,776	17,009
mKate2 silk + Light OFF vs. mKate2 silk + Light ON	-41,733	-2.41	*0.031	-79,607	-3,860

[a)]without green LED light irradiation.
[b)]with green LED light irradiation.

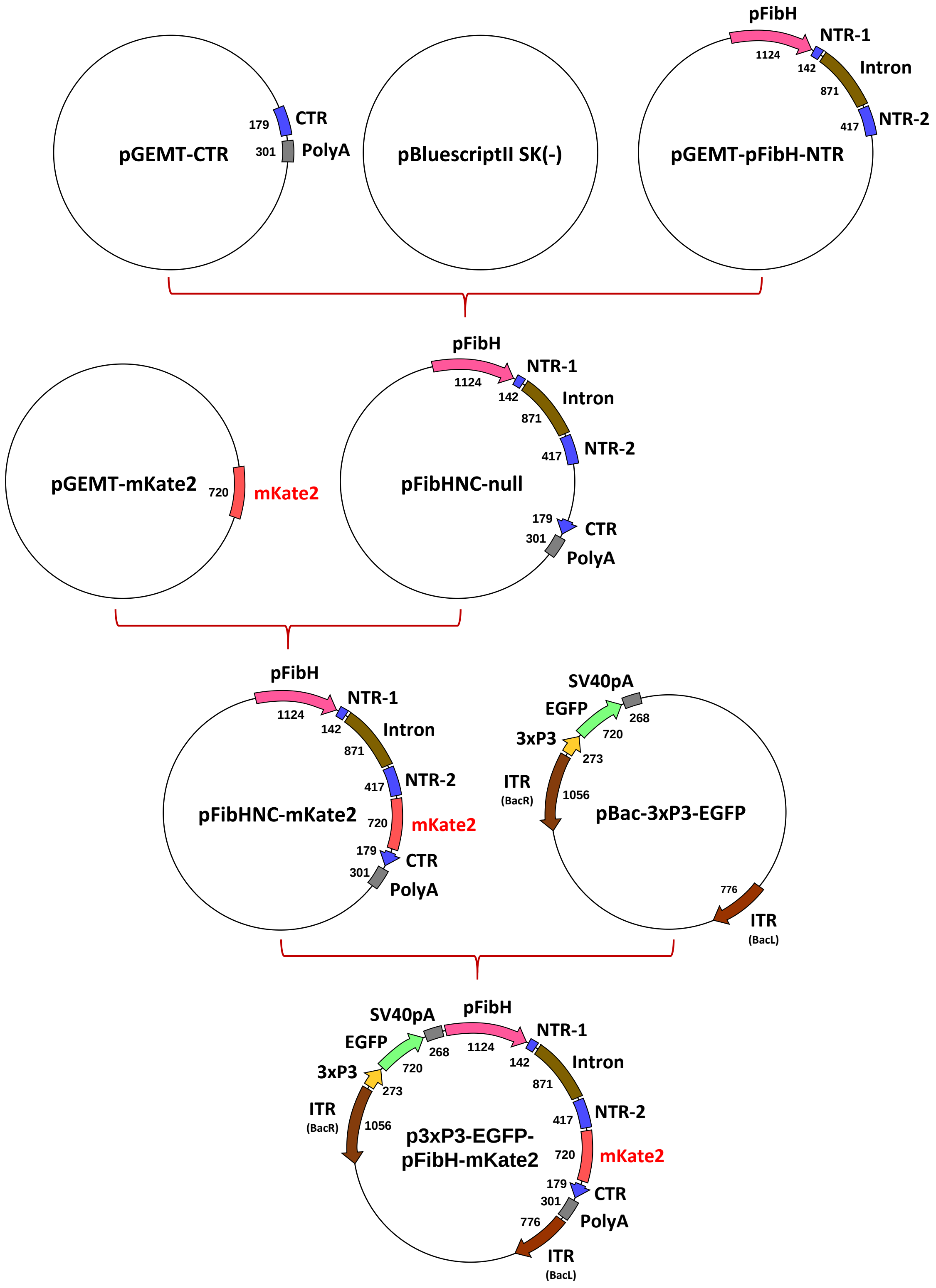

Figure S1. **Construction sequence map of transfer vector p3xP3-EGFP-pFibH-mKate2**. The nucleotide sequences of pFibH-NTR and CTR are derived from Genebank Accession No. AF226688. pFibH: fibroin heavy chain promoter domain (1124 bp), NTR-1: N-terminal region 1 (142 bp), intron: first intron (871 bp), NTR-2: N-terminal region 2 (417 bp), CTR: C-terminal region (179 bp), PolyA: poly(A) signal region (301 bp), EGFP: enhanced green fluorescent protein gene, mKate2: monomeric far-red fluorescent protein, ITR (BacR, BacL): inverted repeat sequences of piggyBac arms, 3xP3: 3xP3 promoter, and SV40: SV40 polyadenylation signal sequence.

a

No	Location	Mr(expt)	Mr(calc)	Sequence
1	49-63	1590.8493	1589.8148	DASGAVIEEQITTKK
2	70-79	2442.8413	2441.1519	NHGILGKNEK
3	83-104	2442.8413	2441.1519	TFVITTDSDGNESIVEEDVLMK
4	159-171	1637.3093	1637.7462	MVSELIKENMHMK
5	172-184	1589.3663	1589.7296	LYMEGTVNNHHFK
6	185-201	1946.4763	1945.8145	CTSEGEGKPYEGTQTMR
7	204-226	2433.9883	2433.1925	AVEGGPLPFAFDILATSFMYGSK
8	252-279	3062.0203	3060.4597	VTTYEDGGVLTATQDTSLQDGCLIYNVK
9	280-294	1660.4443	1660.8242	IRGVNFPSNGPVMQK
10	295-316	2350.9313	2351440	KTLGWEASTETLYPADGGLEGR
11	348-357	1270.9383	1270.6128	MPGVYYVDRR
12	363-379	1973.5673	1973.9330	EADKETYVEQHEVAVAR
13	380-386	881.6313	881.3953	YCDLPSK
14	387-403	1826.5823	1827.8863	LGHRPQQVDSVSYGAGR
15	404-422	1687.4983	1685.7604	GYGQGAGSAASSVSSASSR
16	423-429	945.4853	945.4304	SYDYSRR
17	433-439	843.3973	843.4385	KNCGIPR

b

MRVKTFVILC CALQYVAYTN ANINDFDEDY FGSDVTVQSS NTTDEIIRDA SGAVIEEQIT TKKMQRKNKN
HGILGKNEKM IKTFVITTDS DGNESIVEED VLMKTLSDGT VAQSYVAADA GAYSQSGPYV SNSGYSTHQG
YTSDFSTSAA VGAGSSGRMV SELIKENMHM KLYMEGTVNN HHFKCTSEGE GKPYEGTQTM RIKAVEGGPL
PFAFDILATS FMYGSKTFIN HTQGIPDFFK QSFPEGFTWE RVTTYEDGGV LTATQDTSLQ DGCLIYNVKI
RGVNFPSNGP VMQKKTLGWE ASTETLYPAD GGLEGRADMA LKLVGGGHLI CNLKTTYRSK KPAKNLKMPG
VYYVDRRLER IKEADKETYV EQHEVAVARY CDLPSKLGHR PQQVDSVSYG AGRGYGQGAG SAASSVSSAS
SRSYDYSRRN VRKNCGIPRR QLVVKFRALP CVNCN

N-terminal domain of the fibroin H-chain   C-terminal domain of the fibroin H-chain   mKate2

Figure S2. **Mass spectrometric analyses.** (**a**) Peptides from mKate2. (**b**) Sequence alignment of mKate2/Fibroin H-chain fusion recombinant protein amino acid. The mass density of mKate2/Fibroin H-chain fusion recombinant protein in mKate2 silk is estimated to be ~ 12.6 %.

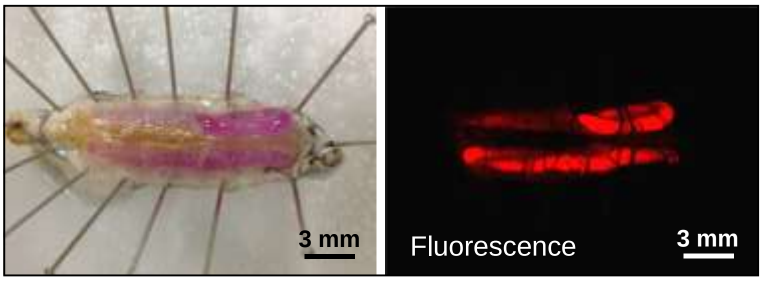

Figure S3. **mKate2 fluorescence in the silk gland of the transgenic line**. Photograph (left) and fluorescent image (right) of the silk gland for the transgenic mKate2 silkworm larvae at the 3rd day of the 5th instar.

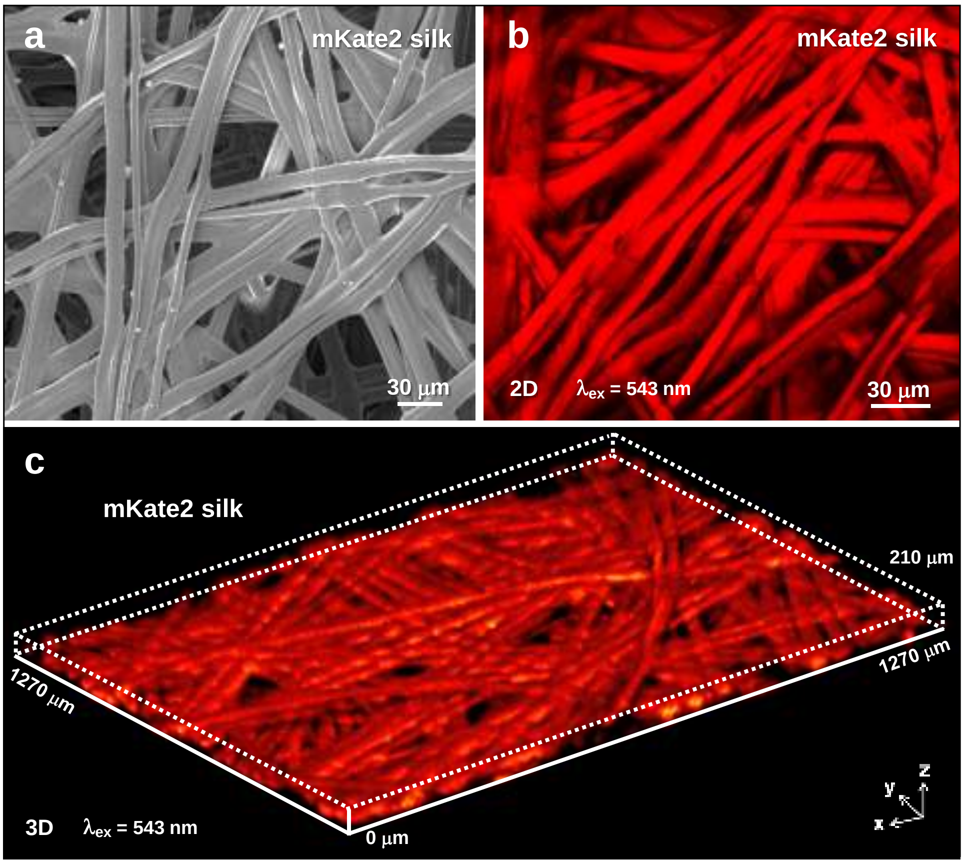

Figure S4. **Microscopic images of mKate2 silk.** (**a**) SEM image of mKate2 silk fibers. (**b&c**) Confocal fluorescence microscopy images of mKate2 silk fibers under green light excitation.

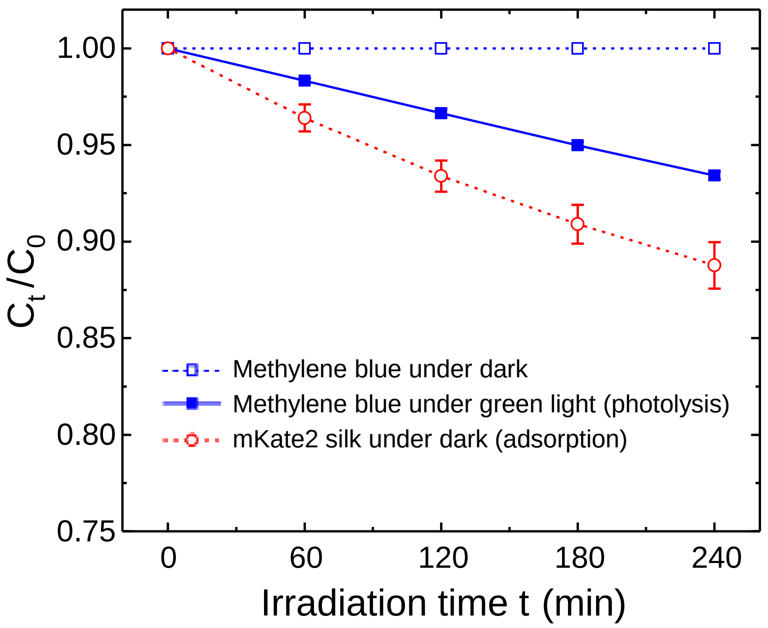

Figure S5. **Confounding factors in photodegradation of methylene blue by mKate2 silk**. The adsorption of methylene blue to mKate2 silk and the photolysis of methylene blue under green light irradiation are separately measured. The error bars are standard deviations.

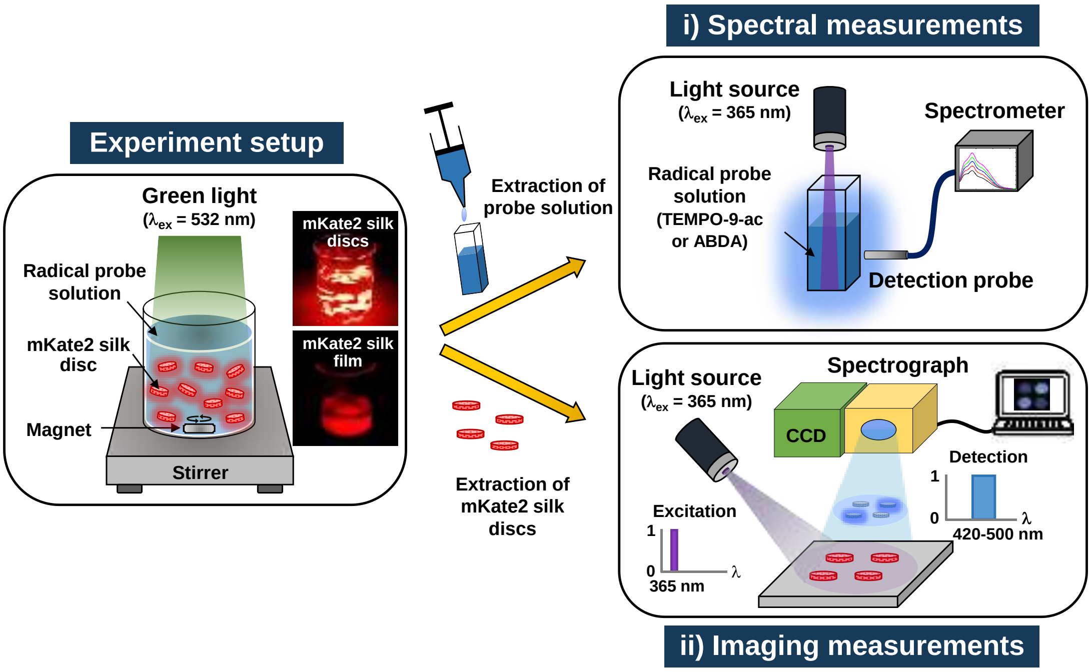

Figure S6. **Two detection scenarios for assessing ROS generated by mKate2 silk upon green light activation**. i) Spectral measurements: Fluorescent signals of radical probes under excitation of λ_{ex} = 365 nm are detected from the solution including mKate2 silk discs. ii) Imaging measurements: Fluorescent radical probes are permeated into mKate2 silk discs. Specimens are arranged within the field of view of the mesoscopic imaging setup, in which the excitation (λ_{ex} = 365 nm) and emission filters (λ_{em} = 420 – 500 nm) are used as illustrated.

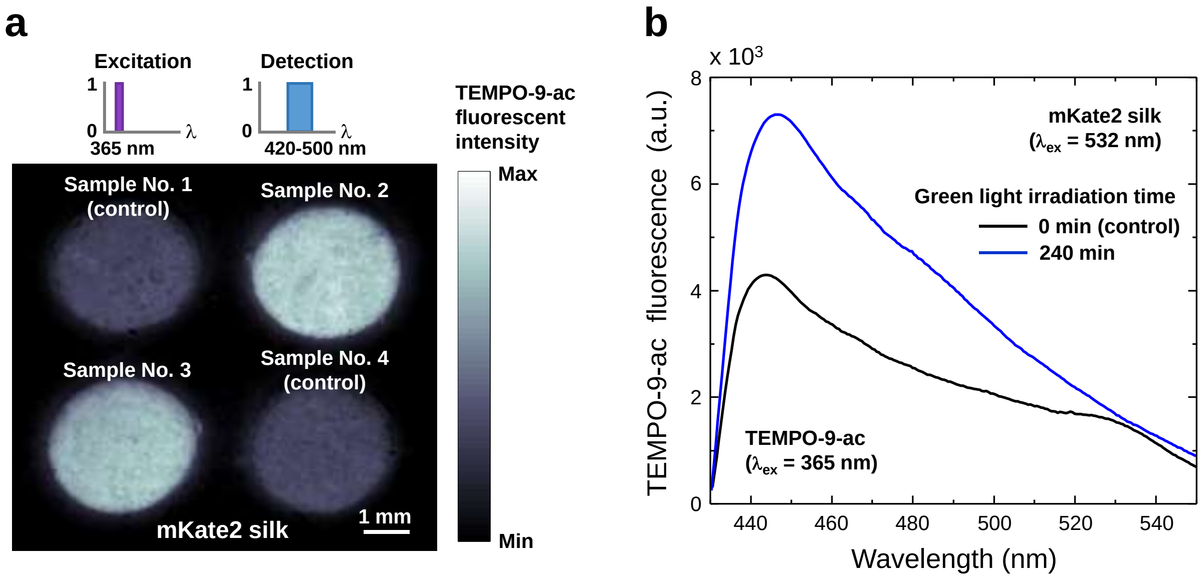

Figure S7. **Turn-on fluorescent signals of TEMPO-9-ac in mKate2 silk.** (**a**&**b**) Fluorescent images (**a**) and spectra (**b**) of TEMPO-9-ac (λ_{ex} = 365 nm) in mKate2 silk discs without green light irradiation (controls) and with green light irradiation for 240 minutes.

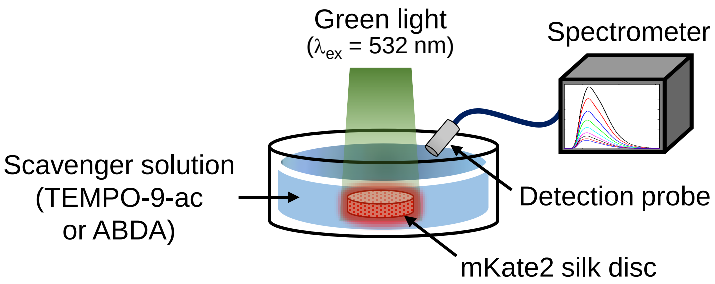

Figure S8. **Photobleaching of mKate2 fluorescence.** TEMPO-9-ac and ABDA, which are used as physical scavengers of phototoxic ROS generated by mKate2 silk, slow down photobleaching of mKate2 in silk.

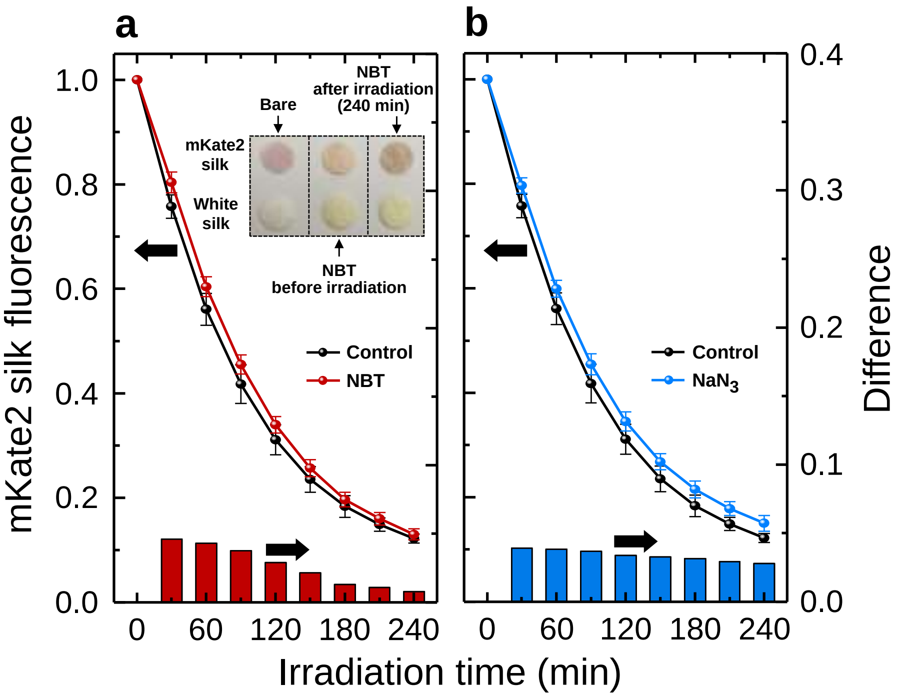

Figure S9. **Scavenger detection of ROS generated by mKate2 silk upon green light activation**. (**a**&**b**) Normalized fluorescent intensity of mKate2 silk with and without scavengers of NBT for $O_2^{\bullet-}$ (**a**) and NaN_3 for $^1O_2$ (**b**), respectively. As a control, the normalized fluorescent intensity of mKate2 silk without the scavengers is plotted in black. The error bars are standard deviations. (Bottom insets) Differences in fluorescent intensity with respect to the control. (Top inset of **a**) Photograph of bare and NBT-treated (before and after light irradiation) white silk and mKate2 silk discs, supporting the $O_2^{\bullet-}$ generation. After 240-minute green light irradiation, there are no variations in the color (yellow) of white silk, while mKate2 silk changes to the bluish color, resulting from the formation of blue chromagen diformazan.

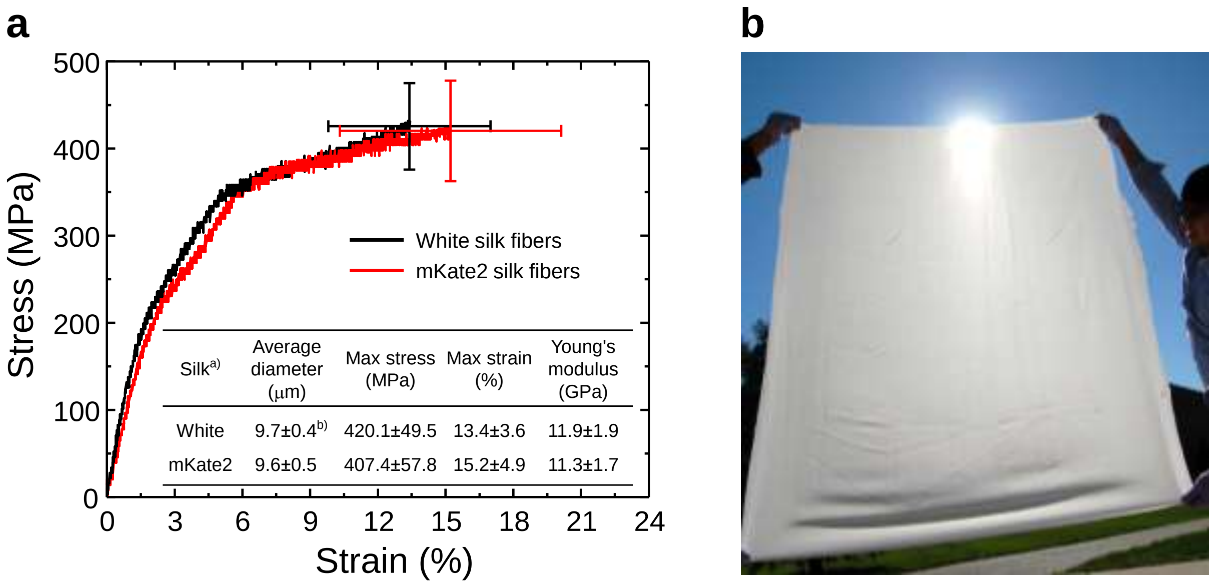

Silk[a)]	Average diameter (μm)	Max stress (MPa)	Max strain (%)	Young's modulus (GPa)
White	9.7±0.4[b)]	420.1±49.5	13.4±3.6	11.9±1.9
mKate2	9.6±0.5	407.4±57.8	15.2±4.9	11.3±1.7

Figure S10. **Mechanical tests of mKate2 silk and scalable/continuous manufacturing of silk fabrics**. (**a**) Representative strain-stress curves of white silk and mKate2 silk fibers. The error bars are standard deviations in the elongation at break (horizontal axis) and the fracture strength (vertical axis). [a)]For each silk, at least 10 randomly selected single fibers from three silk cocoons are tested for statistical analyses. [b)]Mean ± standard deviation. The Young's moduli are calculated from the first linear regime of the strain-stress curve before the first bend. (**b**) Photograph of 110 cm × 140 cm silk fabric woven in the Korea Silk Research Institute (Jinju, Republic of Korea). This white silk fabric did not undergo any additional chemical treatments except for sericin removal (i.e. degumming), showing the possibility of scalable and continuous fabrication using the conventional textile infrastructures.

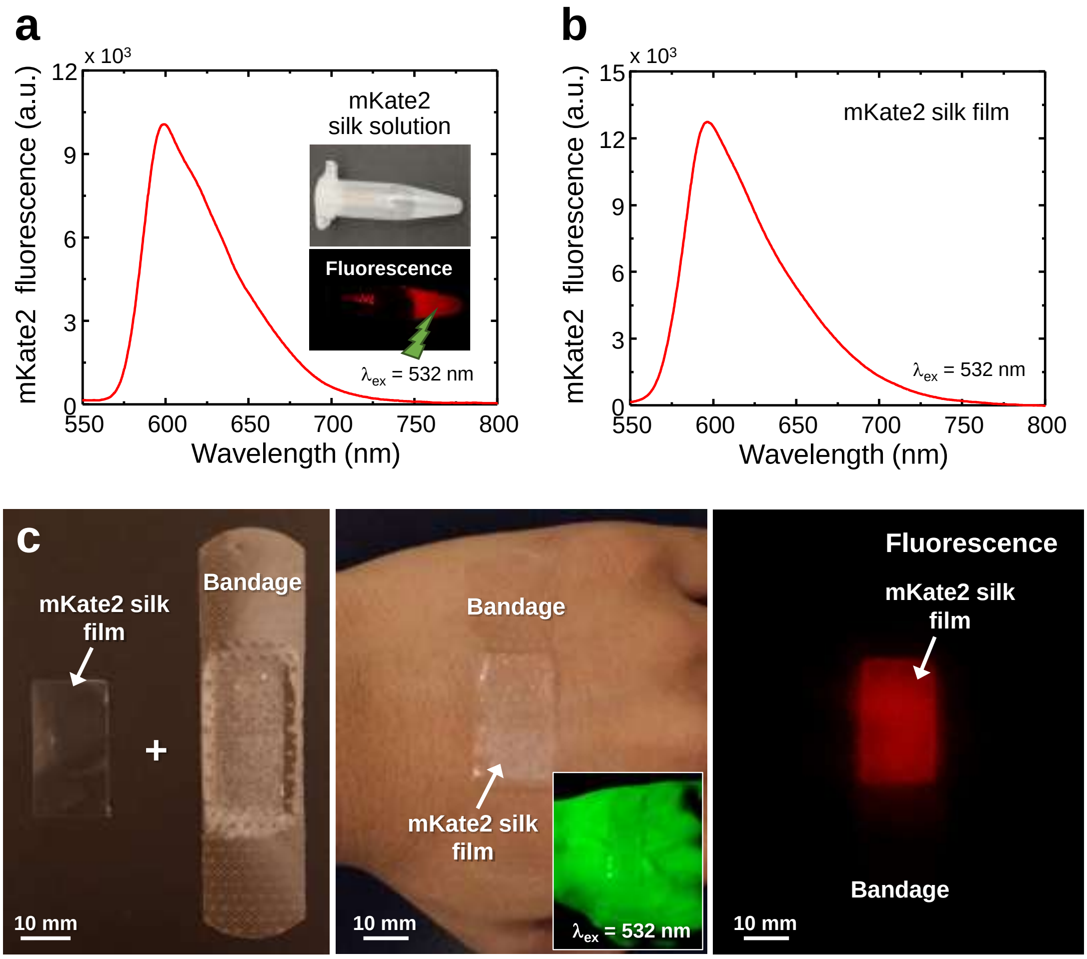

Figure S11. **Fluorescent spectra of regenerated mKate2 silk and representative utilization of regenerated mKate2 silk.** (**a**&**b**) Fluorescent spectra of regenerated mKate2 silk in forms of solution (**a**) and film (**b**), respectively. (Inset of **a**) Photograph and fluorescent image of mKate2 silk solutions. (**c**) A regenerated mKate2 silk film can be integrated with a bandage, potentially offering an additional functionality of controllable ROS release using a simple light source.

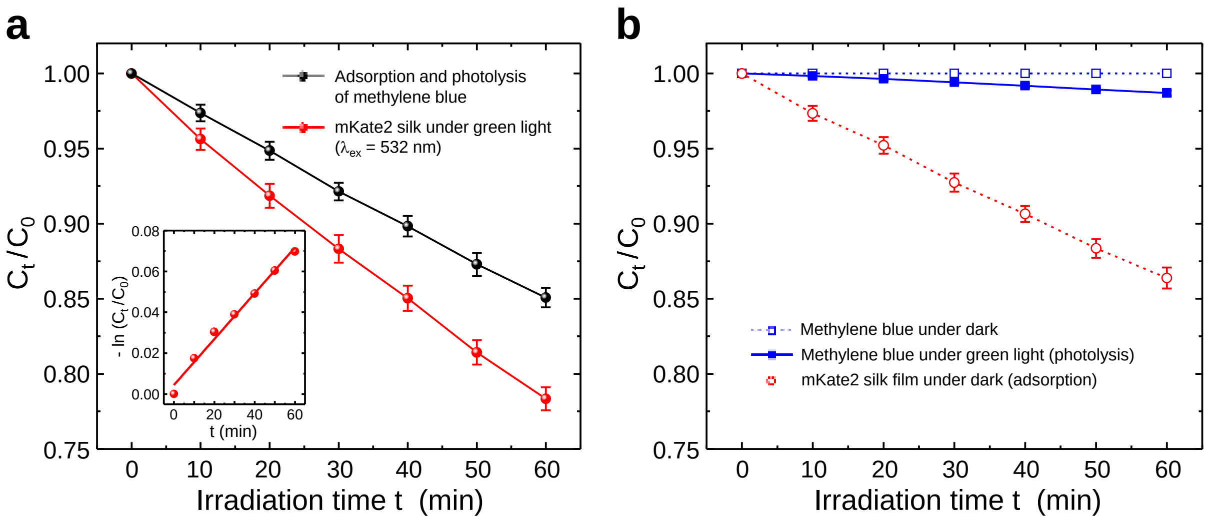

Figure S12. Photocatalytic activity of regenerated mKate2 silk for degrading methylene blue under green light activation at the ambient temperature. (**a**) Photodegradation of methylene blue upon green light activation. (Inset of **a**) Kinetic plot for methylene blue photodegradation by mKate2 silk film after factoring out both adsorption and photolysis of methylene blue (k_{app} = 1.12×10^{-3} min^{-1}). (**b**) Confounding factors in photodegradation of methylene blue by mKate2 silk film for the adsorption of methylene blue to the mKate2 silk film and the photolysis of methylene blue under the green light irradiation. The error bars are standard deviations.
